# Molecular typing methods & resistance mechanisms of MDR *Klebsiella pneumoniae*

**DOI:** 10.3934/microbiol.2023008

**Published:** 2023-02-27

**Authors:** Sunil Kumar, Razique Anwer, Arezki Azzi

**Affiliations:** 1 Department of Microbiology, Kampala International University, Western Campus, Ishaka, Uganda; 2 Department of Pathology, College of Medicine, Imam Mohammad Ibn Saud Islamic University (IMSIU), Riyadh, Saudi Arabia; 3 Department of Biochemistry, College of Medicine, Imam Mohammad Ibn Saud Islamic University (IMSIU), Riyadh, Saudi Arabia

**Keywords:** *Klebsiella pneumoniae*, MLST, molecular typing, PFGE, resistance mechanisms

## Abstract

The emergence and transmission of carbapenem-resistant *Klebsiella pneumoniae* (CRKP) have been recognized as a major public health concern. Here, we investigated the molecular epidemiology and its correlation with the mechanisms of resistance in CRKP isolates by compiling studies on the molecular epidemiology of CRKP strains worldwide. CRKP is increasing worldwide, with poorly characterized epidemiology in many parts of the world. Biofilm formation, high efflux pump gene expression, elevated rates of resistance, and the presence of different virulence factors in various clones of *K. pneumoniae* strains are important health concerns in clinical settings. A wide range of techniques has been implemented to study the global epidemiology of CRKP, such as conjugation assays, 16S-23S rDNA, string tests, capsular genotyping, multilocus sequence typing, whole-genome sequencing-based surveys, sequence-based PCR, and pulsed-field gel electrophoresis. There is an urgent need to conduct global epidemiological studies on multidrug-resistant infections of *K. pneumoniae* across all healthcare institutions worldwide to develop infection prevention and control strategies. In this review, we discuss different typing methods and resistance mechanisms to explore the epidemiology of *K. pneumoniae* pertaining to human infections.

## 1. Introduction

*Klebsiella pneumoniae* is a gram-negative bacterium (GNB) responsible for a significant proportion (4–8%) of nosocomial infections [1]. It is intrinsically resistant to penicillin and often carries some elements for acquired resistance to an array of antimicrobial agents. *K. pneumoniae* has been reported in a variety of infections such as pneumonia, liver abscess, endophthalmitis, and urinary tract infections, even in healthy and young people with competent immune system [2]. *K. pneumoniae* carries a number of extra-chromosomal virulence factors such as salmochelin, aerobactin, heavy metal resistance virulence factors, and capsular polysaccharides [3]. The presence of these virulence factors, which are encoded mostly by plasmids, can be used to distinguish between different strains of *K. pneumoniae* [4]. Carbapenem resistance in *K. pneumoniae* involves manifold mechanisms, such as under-expression or loss of porins, alterations of permeability in the outer membrane, overexpression of efflux transporters along with overproduction of various β-lactamase enzymes or extended-spectrum β-lactamases (ESBLs), or carbapenemase production [5]–[7]. In the United States, *K. pneumoniae* carbapenemase (KPC) is a prevalent serine carbapenemase with high clinical significance and has attracted global attention as a public health threat owing to its rapid international transmission [8],[9].

Over the past two decades, several molecular techniques have been developed for characterizing and typing *K. pneumoniae* strains, including pulsed-field gel electrophoresis (PFGE), repetitive sequence-based PCR (rep-PCR), and multilocus sequence typing (MLST) [10], among which, MLST is considered the gold standard. MLST of *K. pneumoniae* relies on DNA sequence variations in seven housekeeping genes (*rpoB, gapA, mdh, pgi, phoE, infB*, and *tonB*), which together generate a specific allele profile that results in a sequence type (ST) for a single isolate [11]. In MLST, a specific allele number is generated for each distinct gene sequence, followed by the generation of an ST by arranging the seven different alleles in a unique pattern. eBURST (http://eburst.mlst.net/) is another tool used to define isolates that are closely genetically related and assign them to one or more clonal complexes [12],[13].

Ribotyping (16S-23S rDNA ITS [internal transcribed spacer (ITS)] PCR) is a technique with elevated discriminatory power, tremendous reproducibility, and ease of analysis [14]. 16S-23S rDNA ITS PCR for nosocomial infections has been successfully applied to investigate the molecular epidemiology of *K. pneumonia* [15]. Nevertheless, the banding pattern variation and interpretation have both practical and theoretical limitations. A study from Tianjin (China) used PCR detection with 16S-23S rDNA ITS of *K. pneumoniae* to detect the pathogen on infant formula and clinical samples within 48 h [15].

Rep-PCR is a quick and easy tool for investigating hospital outbreaks [16]. This technique was used to investigate the distribution of ESBL genes and molecular typing in Iran [17]. rep-PCR can be used to investigate the epidemic position of multidrug-resistant (MDR) *K. pneumoniae* infections; however, the discriminatory power of this test is absent for a few strains [18]. During an outbreak of sepsis in North India, MDR *K. pneumoniae* isolates were successfully typed by rep-PCR using consensus primers [16].

PFGE is considered the gold standard technique for studying the genetic relatedness and molecular epidemiology of bacterial species [19],[20]. However, in case of *K. pneumoniae*, PFGE may not provide sufficient resolution because of the high clonality of the clinical isolates and the low power of this method to differentiate between clusters. Recently, a modified PFGE protocol was successfully developed to improve the typing of nosocomial isolates of *K. pneumoniae* [21].

MLST is a DNA sequence-based typing technique that provides information on the genetic relatedness and characterization of bacterial isolates [22],[23]. MLST provides portable and unambiguous data, which allows multiple users to obtain information from databases and makes it easy to implement the technique internationally. The MLST scheme is the chief method for studying the evolutionary relationships and characterizing the nosocomial isolates of *K. pneumoniae* [11]. Recently, a study compared the most prevalent typing methods for *K. pneumoniae*, namely, cgMLST, PFGE, and core SNP, and discussed their efficiency in substantiating or eliminating the possibility of nosocomial infection spread [24].

In another recent study, whole-genome sequencing (WGS) has been applied to investigate phylogeny and genetic relatedness and identify *K. pneumoniae* isolates [25],[26]. However, the use of WGS or metagenomics for tracing any local outbreak is limited because of the high cost of conducting the tests and the lack of access to expertise and/or resources. Consequently, conventional methods such as MLST, PFGE, and rep-PCR, along with other cost-effective typing methods, are still used more extensively [27].

In this study, we compiled the available information on various typing techniques of *K. pneumoniae* for investigating the epidemiology, molecular characterization, and tracing of outbreaks in the hospital settings of clinically significant pathogens in acute-care hospitals. This review also discusses how molecular typing methods have enhanced our understanding of *K. pneumoniae* identification, taxonomy, evolution, and genetic relatedness and the transmission of virulence factors and antimicrobial resistance genes.

## 2. Molecular typing of *K. pneumoniae*

### 2.1. Ribotyping (16S-23S ITS)

Ribotyping is a useful tool for studying the epidemiology of different types of pathogenic bacteria [28]. Eight highly conserved operons have been found on the chromosome of *K. pneumoniae*, which are responsible for 16S and 23S rRNA coding. These operons cut with the precise restriction enzyme (RE) and result in restriction pattern bands that are adequate for differentiation and facilitating analyses and interpretations. The greater the choice of restriction enzymes, the greater the discriminatory power of ribotyping [29]. For the ribotyping of various bacterial isolates, an array of REs have been used. Several investigators have recommended that the power of discrimination of ribotyping can be improved by using more than one RE. Ribotyping typeability has been shown to rely on the REs utilized. Epidemiological studies on *K. pneumoniae* are inadequate because of the absence of a reliable, single, and convenient epidemiological typing scheme. To investigate any *K. pneumoniae* outbreak, ribotyping has proven to be an outstanding typing method with immense discriminatory power [30]. Other conventional typing techniques have several limitations such as typeability, inadequate reproducibility, and insufficient discriminatory power [10]. To circumvent these limitations, more typing methods that use molecular techniques are being employed [31]. Using a digoxigenin-labeled rDNA probe and *EcoRI* RE, a ribotyping database of *K. pneumoniae* was created without the harmful effects of radioactive probes [28].

### 2.2. PCR-based replicon typing (PBRT)

To investigate any suspected bacterial outbreak, a swift molecular typing scheme can be an excellent tool. PCR-based replicon typing (PBRT) is a simple and rapid method for investigating outbreaks of nosocomial pathogens [16]. This technique was invented for *Enterobacteriaceae* plasmids based on the *repA* gene, which categorizes plasmids into different incompatible (Inc) groups [32]. One study investigated PCR with the following plasmid types: *IncFII, IncFIA, IncFIIK, IncFIB, IncHI1B, IncR, IncN, IncL/M*, *IncA/C,* and *IncB/O* by PBRT in India [33]. That study suggested the transfer of antimicrobial resistance (AMR) genes using a variety of plasmids. Some of these limitations have been resolved by developing a categorization scheme derived from the identification of basic replicons using DNA hybridization and a PBRT technique facilitating extensive plasmid typing [34]. For many decades, plasmid classification has been a significant spotlight for plasmid biologists because of their role in animal and human health, microbial evolution and adaptation, and environmental processes.

### 2.3. Rep-PCR

Rep-PCR is a typing method that distinguishes microbes by amplifying DNA fragments composed of sequences between repetitive elements in the presence of complementary primers that complement interspersed repetitive consensus sequences. Amplicons of different sizes can be fractioned using electrophoresis, and the resulting DNA fingerprint patterns can be compared with those specific to individual bacterial clones. Numerous studies have shown that rep-PCR using primers derived from repetitive extragenic palindromic (REP) elements (REP-PCR) or enterobacterial repetitive intergenic consensus (ERIC-PCR) sequences is effective in typing a wide variety of bacteria. In a study from Denmark, a semi-automated rep-PCR typing technique was used to reveal the association between the recognized outbreak strains and ESBLs produced by local *K. pneumoniae* strains [35].

### 2.4. Pulsed-field gel electrophoresis (PFGE)

To perform epidemiological investigations and trace genetic relatedness, PFGE is considered the gold standard for many bacterial species [19],[20]. However, this technique may not be adequate for discriminating between different clusters because of the high clonality of clinical isolates of *K. pneumoniae*, thus failing to discriminate the transmission dynamics. Recently, a modified PFGE method has been devised for improving the typeability of nosocomial isolates of *K. pneumoniae* [21]. Carbapenemase-producing *K. pneumoniae* strains were recently analyzed for molecular characterization using PFGE in Turkey, which demonstrated the presence of 24 pulse types, and 63.09% of isolates were represented by four main pulse types [36]. This was the first event of coproduction of the two genes (bla_OXA-48 + KPC_ and bla_KPC + NDM_) in carbapenem-resistant *Klebsiella pneumoniae* (CRKP) isolates from Turkey. The diversity and heterogeneity of *mcr-8* were investigated in chicken-related *K. pneumoniae* in China using S1 nuclease PFGE (S1-PFGE), which showed that the *mcr-8* (mobile colistin resistance) gene was located on a plasmid in all isolates [37]. The findings of that study indicated that the heterogeneous and diverse genetic context of *mcr-8* is responsible for the increased level of colistin resistance in *K. pneumoniae* isolates. In a study from South India, molecular insights into carbapenem resistance were investigated in the clinical isolates of *K. pneumoniae* that targeted MDR using PFGE [38]. That study reported a total of 16 diverse PFGE patterns in 18 MDR isolates of *K. pneumoniae*. Another study from North India conducted genetic characterization of CRKP clinical isolates using S1-PFGE and MLST and suggested massive plasticity of the genome of *K. pneumoniae* isolates, showing the potency to transmit antimicrobial resistance. In the northern region of Portugal, the molecular epidemiology of 106 clinical isolates of carbapenemase-producing *K. pneumoniae* was investigated using PFGE for the first time [39]. A total of 29 PFGE types were identified in that study. ESBL-producing *K. pneumoniae* colonizing the gastrointestinal tract were typed using PFGE and confirmed to have high genetic variation in patients in a cancer hospital in Poland [40]. The polyclonal spread of colistin-resistant *K. pneumoniae* was studied using PFGE in a Croatian hospital and outpatient setting, and strains belonging to six PFGE clusters were identified [41].

### 2.5. Multilocus sequence typing (MLST)

MLST is a DNA sequence-based technique suitable for molecular characterization and genetic relatedness of many bacterial genera [22],[23],[42]. MLST provides unequivocal and transferable data, enabling the execution of evolutionary analyses by multiple users using global databases. An MLST scheme was developed to characterize and analyze *K. pneumoniae* nosocomial isolates [11]. Recently, homology analysis was performed using MLST for clinical isolates of enteral and extraintestinal *K. pneumonia* among neonates in China, with six sequence types [43]. A total of 74 carbapenemase-resistant isolates of highly virulent *K. pneumoniae* were investigated in the Zhejiang Province of China using MLST to explore the clinical features of patients with diverse sequence types of infections [23]. A total of 17 ST types were allocated to 74 isolates, with ST 11 being the dominant one showing elevated resistance to 21 frequently used antimicrobial agents. In India, 290 CRKP isolates from seven different centers were investigated using MLST [44]. A total of 75 diverse STs were identified in the latter study, of which ST231 was the most common. XDR hypervirulent *K. pneumoniae* was typed in another study from India to investigate neonatal sepsis in a tertiary care hospital in India [45]. All isolates were typed using MLST and belonged to ST5235. A study from Taiwan showed the molecular epidemiology of 43 *K. pneumoniae* isolates using PFGE, which revealed the transmission of several clones [46]. Six of the 12 tested *K. pneumoniae* representatives of different pulsotypes belonged to IncA/C. KPC-2-producing *K. pneumoniae* isolates from bovine mastitis in Mexico were characterized using MLST, which showed that all isolates comprised two clones belonging to ST258 [47]. Phylogenetic analysis of *K. pneumoniae* ST340 strains using MLST revealed the presence and distribution of *bla*NDM-5 in porcine pneumonia isolates from China [48]. This study demonstrated an MDR profile for a broad spectrum of antibiotics, including gentamicin, meropenem, ciprofloxacin, various cephalosporins, azteonam, and florfenicol. The first report on the clonal relationship among KPC-2-producing CRKP strains was performed using MLST in the Department of Urology at Annaba Hospital, Algeria [49]. MLST revealed two dissimilar STs of 14 *K. pneumoniae* isolates, namely, ST101 and ST258.

### 2.6. Matrix-assisted laser desorption ionization-time of flight mass spectrometry

Currently, MLST is used for molecular characterization and epidemiological investigations, which is quite expensive, arduous, and time-consuming [50]–[52]. As an alternative, substitute typing schemes such as matrix-assisted laser desorption ionization-time of flight mass spectrometry (MALDI-TOF MS) has been studied [52]–[54]. A study revealed that MALDI-TOF MS could be utilized as an alternative method for molecular typing of CRKP isolates that produce carbapenemases [55]. An outbreak of KPC-3-producing *K. pneumonia* that resulted in an interhospital spread in Spain was determined using the analytical efficiency of MALDI-TOF [56]. Additionally, quick detection of KPC-harboring *K. pneumoniae* in China and India has been performed using MALDI-TOF MS [51],[57]. Recently, a web-based tool, “Klebsiella MALDI Type R,” was developed as a user-friendly and platform-independent application that facilitates the uploading of MS data of MALDI-TOF, with the aim of identifying Klebsiella strains at the phylogroup and species levels [58]. The molecular epidemiology and clinical features of CRKP infections in central China were assessed using MALDI-TOF [59]. A total of 71 isolates were observed with 11 mass spectrometry (MS) types, of which 38 (53.5%) were MS4 or MS6. The discriminatory power of MALDI-TOF MS, along with two other typing techniques, was assessed in South India to determine the genetic diversity of nosocomial isolates of *K. pneumoniae* from a tertiary hospital [60]. The MALDI-TOF system was used in Italy for the real-time identification of KPC production and typing of *K. pneumoniae* clinical isolates [12]. Using MALDI-TOF MS, rapid identification and clustering of *K. pneumoniae* isolates were performed from different restaurant sites in the Al-Qassim region of Saudi Arabia [61]. MALDI-TOF MS has also been used for the quick identification and typing of many other bacterial species [62].

### 2.7. Whole-genome sequencing (WGS)

WGS is considered the most powerful technique for monitoring and exploring the epidemiology of *K. pneumoniae* by creating an all-inclusive representation of bacterial populations in one assay. It facilitates instantaneous identification of all bacterial species, resistance, lineage, and virulence determinants [63],[64]. Many blood isolates of CRKP from Israel have been explored using WGS to reveal the mechanisms of colistin resistance [65]. WGS is quickly gaining attention as a key method for surveillance and epidemiological investigations of *K. pneumoniae* because of its ability to reveal the complexity and genetic relatedness of the bacterium. WES-based typing and phylogenetic analysis of 39 randomly chosen geographically dissimilar MDR *K. pneumoniae* isolates were performed in nine hospitals in Egypt [66]. Notably, the data generated by WGS surveillance are used for multiple purposes such as studying local disease epidemiology and understanding the continuing evolution and transmission of clinically significant strains [67],[68]. WGS of CRKP has also resulted in extensive detection of the function of mobile genetic elements and plasmids in hospital outbreaks, where *K. pneumoniae* receives and donates the *AMR* gene in a closed environment [68]–[70].

### 2.8. Infrared biotyping (IRBT)

Recently, in China, *K. pneumoniae* isolate typing was evaluated using the IR Biotyper (IRBT) technique (a Fourier transform infrared [FTIR] spectroscopy system) for its potential use in hospital hygiene management via (i) standardizing the culture methods and describing the cutoff value limits and (ii) evaluation with frequently used typing methods such as MLST, WGS, and PFGE [71]. The typing results of IRBT were almost entirely concordant with those obtained using PFGE and WGS. Together with its merits such as cost-effectiveness and less time consumption, IRBT is an efficient technique for typing bacterial strains, which could be used reliably for the real-time investigation of hospital outbreaks [71].

## 3. Resistance mechanisms in *K. pneumoniae*

### 3.1. Acquired resistance

Different determinants of antibiotic resistance are encoded chromosomally, which are capable of increasing the resistance against various antimicrobial agents in *K. pneumoniae*; a few of these have migrated to other bacterial species [72]. Fosfomycin resistance genes fosA, SHV beta-lactamase, and the nalidixic acid efflux transporter OqxAB are examples of such resistance determinants in *K. pneumoniae*. Therefore, tracing the genes that promote the typical resistant phenotype is a fascinating area to facilitate the prediction of future bacterial evolution and detect recognized *AMR* genes whose transmission gives antimicrobial resistance to different hosts.

### 3.2. Intrinsic resistome

Recently, many studies have been performed to detect genes responsible for specific antimicrobial susceptibility among various pathogens, known as the intrinsic resistome. The intrinsic resistome is defined as “the ensemble of chromosomal genes that are involved in intrinsic resistance and whose presence in strains of a bacterial species is independent of previous antibiotic exposure and is not due to horizontal gene transfer.” The investigation of transposon-tagged libraries can provide information on two facets of antimicrobial resistance. The activation of a few genes enables bacteria to become more susceptible to antimicrobial agents. Such genes play a significant role in fostering the true intrinsic resistome, as their existence enables bacteria to become more resistant to antibiotics and might be a good target for various inhibiting agents in combination with antimicrobials. Additionally, the transmission of these genes may contribute to the resistance phenotypes of a different host. The second group includes genes whose inactivation diminishes antimicrobial susceptibility. Resistance-derived mutations could be predicted using this group of genes. In a recent study, such mutants were discovered in a jumping gene-tagged library in *K. pneumoniae* strains [73].

### 3.3. Carbapenem resistance

Carbapenem is believed to be one of the most efficient antimicrobials for treating serious bacterial infections. However, resistance to this class of drugs has been reported in some cases, which is an alarming concern for public health [74]. Resistance to carbapenems is a global health issue that occurs primarily in GNB pathogens, such as *Pseudomonas aeruginosa, Acinetobacter baumannii*, and *K. pneumoniae* [75]–[77]. Acquired or intrinsic resistance mechanisms may be responsible for the failure of carbapenem treatment. Modifications in the porins of bacterial cell result in reduced levels of uptake of β-lactam drugs mainly in GNB [77],[78]. This reduces the permeability of the outer membrane, which prevents the drug from binding to its target in the bacterial cell [79]. There are many other acquired mechanisms of resistance in bacteria, including efflux pumps, alteration of the target site, and enzymatic degradation of drugs [80],[81]. A significant number of ESBL genes have the capability to get transmitted among different bacteria [82]. In contrast, strains with altered expression of their porins characteristically do not show the capacity for transmission but only propagate in the hospital environment. *K. pneumoniae* have been observed to portray such kind of mechanisms [83]. In *Klebsiella* species, a reduction in porins and overexpression of efflux transporters are the leading causes or mechanisms of resistance against imipenem drugs [83],[84].

#### 3.3.1. Classification of carbapenemase enzymes

##### 3.3.1.1. Class A carbapenemases

KPCs (KPC-2–KPC-13) are the most common carbapenemases found in *K. pneumoniae* [85]. Immediately after its identification, KPCs spread worldwide and resulted in many outbreaks in Africa, Asia, European countries, and North America [86]–[89]. Bacteria-producing KPCs have progressed and become resistant to multiple antimicrobials, thus limiting treatment choices for managing infections [90],[91].

##### 3.3.1.2. Class B carbapenemases

These classes include carbapenem-hydrolyzing enzymes, which are classified as the chief class of β-lactamases but are inhibited by EDTA, a chelator of Zn^2+^ ions and additional divalent cations. β-lactam drug interaction with zinc ions is behind the mechanism of hydrolysis in the active site of the β-lactamase [92]. Different types of integrons and gene cassettes harbor genes that encode carbapenem-hydrolyzing enzymes [93]. NDM-1 (New Delhi metallo-β-lactamase) is the most frequently reported metallo-β-lactamase enzymes including [94], VIM (verona integron-encoded metallo-β-lactamase), IMP (imipenem-resistant pseudomonas) type carbapenemase, SIM (Seoul imipenemase), and GIM (German imipenemase). NDM coding genes are dominant in *K. pneumoniae* isolates [95],[96].

##### 3.3.1.3. Class D carbapenemases

These are serine-β-lactamases enzymes that are weakly inhibited by clavulanic acid or EDTA. These enzymes show poor activity against carbapenems and are often known as OXA-type enzymes [97]. OXA-48 is the most prevalent and widely spread class D β-lactamase in *K. pneumoniae* in Turkey, the Middle East, Europe, and North Africa [98]. This class of carbapenemases is also prevalent in *A. baumanni* clinical isolates [78].

### 3.4. Fosfomycin resistance

Fosfomycin is a potent therapeutic agent for treating *K. pneumoniae* severe infections. The *uhpT*, *fosA,* and *glpT* genes play a crucial role in conferring fosfomycin resistance in *K. pneumoniae* isolates [99]. Fosfomycin causes bacterial cell death by disrupting the biosynthesis of peptidoglycans via inhibition of the MurA enzyme (UDP-NAG-3-enolpyruviltransferase). *FosA* may be present on either a plasmid or a chromosome, resulting in high copy-number plasmids, which give rise to escalated resistance to fosfomycin [100],[101]. Fosfomycin enters the bacterial cell through membrane porins glycerol-3-phosphate transporter (GlpT) and hexose phosphate transporter (UhpT) [102]. The metallo-glutathione *S*-transferase enzyme is encoded by *fosA*, which is extensively distributed in the genomes of *K. pneumoniae* and is also found in other GNB.

For the first time, in a Turkish hospital, *K. pneumoniae* urine isolates showed fosfomycin resistance due to the co-existence of two genes, *blactx-m* and *fosA3* [103]. A study in Azerbaijan conducted an epidemiological investigation of drug resistance in the clinical isolates of *K. pneumoniae* [66]. This study showed a high prevalence of *fosA* (40%), followed by *fosX* (40%) and *fosC* (20%), which confer fosfomycin resistance [104]. However, the distribution of *K. pneumoniae* phylogenetic groups and their association with antibiotic resistance patterns showed the highest sensitivity to fosfomycin (85%) in Tabriz, Iran [105]. A recent study from Wenzhou, China, revealed *fosA3* as the key mechanism of fosfomycin resistance in CRKP isolates, which can spread widely in hospitals through plasmids. Mutations in *glpT* and *murA* have been detected in *fosA3*-negative fosfomycin-resistant CRKP isolates [106]. The rate of fosfomycin resistance in *K. pneumoniae* was found to be increased three-fold in a study conducted in 2020 on urine samples from emergency departments of different hospitals in France [107].

### 3.5. Mechanisms of colistin (polymyxin) resistance

Over the past few decades, colistin resistance has increased in *K. pneumoniae* isolates, which is conferred by several mechanisms. The prevalence of colistin resistance in clinical isolates of *K. pneumoniae* was recently investigated using genomic sequencing [108]. The T246A substitution of amino acids in *PmrB* is the most frequent chromosomal mutation coupled with colistin resistance, which was detected in the most resistant isolates of *K. pneumoniae* (85%) in a recent study from China [108]. A similar substitution was reported in a teaching hospital in Tunisia, in which most *K. pneumoniae* isolates were resistant to colistin antibiotics [109]. Gene expression in the PhoP/PhoQ system is upregulated by mutations in *mgrB*, which also leads to the augmentation of colistin resistance in *K. pneumoniae*. *MgrB* mutations commonly originate from insertion sequences [110]. The phosphorylation of lipid A in the lipopolysaccharide of *K. pneumoniae* is affected by mutations in *phoPQ, pmrAB, mgrB*, and *crrAB* through the pmr-HFIJKLM cluster, which results in polymyxin resistance. Polymyxin resistance is also conferred by the induced and mobile *mcr* gene, overexpression of efflux pumps, and downregulation of porins and membrane-spanning protein *ecr* [111]. The positive charge is augmented by such modifications of membrane lipopolysaccharides, which alter and reduce colistin binding, conferring colistin resistance [112]. In addition, the overproduction of capsular polysaccharides decreases the colistin activity on the surface of bacterial cells and hence imparts colistin resistance [112]. Additionally, *mcr* plays a crucial role in the distribution of colistin resistance among different bacteria via horizontal gene transfer [113],[114]. To date, nine variants of novel *mcr* have been identified. There are still some unknown molecular mechanisms for colistin resistance, which necessitate further exploration.

### 3.6. Extended-Spectrum Beta-Lactamase (ESBL)

Over the last 15 years, numerous outbreaks of infections with organisms producing ESBLs have occurred worldwide [115]. Because of the advent of ESBL producers, many classes of antibiotics, most notably cephalosporins, are threatened by the emergence of ESBLs. Several studies have indicated that poor outcomes can occur in patients with serious infections caused by ESBL-producing organisms that are treated with antibiotics to which the organism is resistant in order to treat the infection [116]. Several species of *K. pneumoniae* have been reported to produce ESBLs in the past, including *K. pneumoniae* strains first discovered in Germany in 1983; since this time, increased resistance to cephalosporins has been observed. Several types of ESBLs are encoded by transferable conjugated plasmids that usually encode antibiotic resistance [117]. ESBLs are capable of readily hydrolyzing penicillin and cephalosporins but have a much lower affinity for cephamycins and clavulanates, which are encoded by various gene variants. For the molecular detection of ESBL genes, a number of groups have been used, including TEM (Temoniera), CTX-M (cefotaximase-Munich), SHV (sulfhydryl variable), and OXA (oxacillin), all of which are categorized as major groups [118],[119]. In recent years, intensive use of cephalosporins has been strongly associated with resistance to ESBLs and outbreaks [120]. Recently, ESBLs and AmpC b-lactamases have become increasingly prevalent in clinical settings. These ESBLs are most commonly produced by *Klebsiella* species but may also occur in other GNBs.

## 4. Conclusion

Molecular typing is important for monitoring infection control in healthcare facilities. Various methods are available for molecular typing and variability. However, genomics has revealed outstanding genetic diversity, which has considerably enhanced *K. pneumoniae*, perceptive pathogenicity, antimicrobial resistance, and diffusion in hospital settings. Molecular methods are crucial for obtaining important information on resistance transmission through the spread of clonal complexes worldwide. Molecular typing and other typing methods can also be used to determine the specific mechanisms of resistance against specific antimicrobial agents. Combining random population surveys with WGS-based genomic analyses could prove to be a dominant approach for detecting and authenticating further clinical genomic markers. However, cost-inclusiveness is an issue when utilizing WGS in survey-based studies, and small-scale investigations can be beneficial.
